# The Genetic Differentiation of *Pyrrhulina* (Teleostei, Characiformes) Species is Likely Influenced by Both Geographical Distribution and Chromosomal Rearrangements

**DOI:** 10.3389/fgene.2022.869073

**Published:** 2022-05-04

**Authors:** Pedro H. N. Ferreira, Fernando H. S. Souza, Renata L. de Moraes, Manolo F. Perez, Francisco de M. C. Sassi, Patrik F. Viana, Eliana Feldberg, Tariq Ezaz, Thomas Liehr, Luiz A. C. Bertollo, Marcelo de B. Cioffi

**Affiliations:** ^1^ Laboratório de Citogenética de Peixes, Departamento de Genética e Evolução, Universidade Federal de São Carlos, São Carlos, Brazil; ^2^ Laboratório de Genética Animal, Coordenação de Biodiversidade, Instituto Nacional de Pesquisas da Amazônia, Manaus, Brazil; ^3^ Institute for Applied Ecology, University of Canberra, Canberra, NSW, Australia; ^4^ Institute of Human Genetics, Friedrich Schiller University, University Hospital Jena, Jena, Germany

**Keywords:** fishes, neo-sex chromosomes, chromosomal rearrangements, cytogenetic, genetic diversity

## Abstract

Allopatry is generally considered to be one of the main contributors to the remarkable Neotropical biodiversity. However, the role of chromosomal rearrangements including neo-sex chromosomes for genetic diversity is still poorly investigated and understood. Here, we assess the genetic divergence in five *Pyrrhulina* species using population genomics and combined the results with previously obtained cytogenetic data, highlighting that molecular genetic diversity is consistent with their chromosomal features. The results of a principal coordinate analysis (PCoA) indicated a clear difference among all species while showing a closer relationship of the ones located in the same geographical region. This was also observed in genetic structure analyses that only grouped *P. australis* and *P. marilynae*, which were also recovered as sister species in a species tree analysis. We observed a contradictory result for the relationships among the three species from the Amazon basin, as the phylogenetic tree suggested *P. obermulleri* and *P. semifasciata* as sister species, while the PCoA showed a high genetic difference between *P. semifasciata* and all other species. These results suggest a potential role of sex-related chromosomal rearrangements as reproductive barriers between these species.

## Introduction

The Neotropical region harbors most of the known fish diversity, with more than 5,200 valid species, however, there is additional evidence that many of them are yet to be described (Fricke et al., 2021). Several reasons are suggested to be related to this huge diversity, and among them, the evolution of reproductive barriers reducing gene flow plays a key role in creating reproductive incompatibilities between species (Mayr, 1963). The evolution of strong reproductive barriers is essential for coexistence of species, especially for sympatric ones (those whose geographical distribution overlaps), even if the isolation in sympatry has preceded the overlap of species that currently coexist in the same region, in a secondary contact (Mayr, 1999; Coyne and Orr, 2004).

Among animals, most cases of post-zygotic isolation are caused by genetic incompatibilities, among which chromosomal rearrangements including specific sex chromosome-systems are of fundamental importance (Bateson, 1909; Dobzhansky, 1933; Muller, 1942; Coyne and Orr, 2004). Such chromosomal rearrangements can prevent introgression and reduce gene flow by suppressing recombination (Machado et al., 2007; Yannic et al., 2009; McGaugh and Noor, 2012; Ostberg, et al., 2013; Presgraves, 2018; Potter, et al., 2021). However, the contribution of these processes for biodiversity in Neotropical fish species is still poorly understood.

The Lebiasinidae family is distributed throughout Central and South America, with 75 species divided into two subfamilies: Lebiasininae and Pyrrhulininae (Weitzman and Weitzman, 2003; Froese and Pauly, 2014; Fricke et al., 2021). The subfamily Pyrrhulininae is the most diverse, including the genera *Derhamia, Nannostomus, Copella, Copeina,* and *Pyrrhulina* (Fricke et al., 2021). Recent cytogenetic and molecular data have added significant contributions to understanding evolutionary relationships of these genera (Moraes et al., 2017, 2019, 2021; Sassi et al., 2019; Toma et al., 2019; Sember et al., 2020). Particularly, concerning *Pyrrhulina*, its diploid number (2n) ranges from 32 to 42 chromosomes, with *Pyrrhulina* aff. *australis*, *P. australis* (Eigenmann and Kennedy, 1903), *Pyrrhulina* aff. *marilynae* and *Pyrrhulina* sp. present 2n = 40 chromosomes, whereas *P. brevis* (Steindachner, 1876), *P. obermulleri* (Allen, Meyers and Myers, 1926)*,* and *Pyrrhulina* cf. *laeta* have 2n = 42 chromosomes, both in males and females. Differently from the others, *P. semifasciata* (Steindachner, 1876) has divergent 2n among sexes (2n = ♀42 and ♂41) due to its exclusive multiple ♀X_1_X_1_X_2_X_2_/♂X_1_X_2_Y sex chromosome system (Moraes et al., 2017; Moraes et al., 2019). In addition, *P. marilynae* (Netto-Ferreira and Marinho, 2013) also differs from its congeneric species having 2n = 32 chromosomes, due to a series of chromosomal fusions (Moraes et al., 2021).

The development and improvement of large-scale genotyping-by-sequencing (GBS) procedures allowed high-resolution analysis using Single Nucleotide Polymorphisms (SNPs) for genetic diversity studies in non-model organisms (Steane et al., 2011; Yang et al., 2013; Sánchez-Sevilla et al., 2015; Alkimin et al., 2018; Souza et al., 2019; Oliveira et al., 2020; Sassi et al., 2021). Combining such genomic and cytogenetic data for pairs of species/populations in which there are variations related to geographic distribution, analysis of chromosomal rearrangements, and sex chromosome systems provide a critical tool to understanding the role of these events to generate biodiversity. The present study applies cytogenetic and genomic approaches in five *Pyrrhulina* species with distinct geographic distributions. Here, we aimed to assess the genetic diversity in five *Pyrrhulina* species by combining population genomic with previously obtained cytogenetic data. Our results highlighted that genomic diversity is consistent with the chromosomal features and provided hypothesis on the potential role of chromosomal rearrangements, especially those involving the sex chromosomes, in fostering genetic differentiation.

## Materials and Methods

### Sampling

**FIGURE 1:**
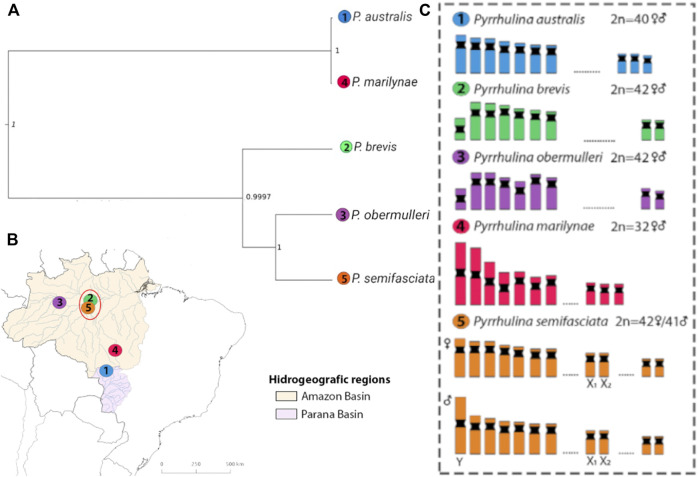
Phylogenetic relationships of *Pyrrhulina* species analyzed and their distribution. **(A)**—Species tree recovered with SNAPP, with branch lengths measured in units of expected number of mutations per site, based on dataset I: *P. australis* (1), *P. brevis* (2), *P. obermulleri* (3); *P. marilynae* (4); and *P. semifasciata* (5). **(B)**—South America map indicating the origin of *Pyrrhulina* species analyzed. Colored areas indicate the distribution of the species: *P. australis* (blue), *P. brevis* (green), *P. obermulleri* (yellow); *P. marilynae* (purple); and *P. semifasciata* (pink). The numbered circles indicate the collection sites of each species. The red ellipse indicates syntopic species (sampled in the same area). **(C)**—Idiograms with partial karyotypes of each species and the sex chromosomes exclusively found in *P. semifasciata* are boxed.

Collection sites, number, and sex of the specimens are given in Figure 1; Table 1. Sampling authorizations were obtained from the Brazilian Environmental Agency, Instituto Chico Mendes de Conservação da Biodiversidade/Sistema de Autorização e Informação em Biodiversidade (ICMBio/SISBIO - license no. 48628-14) and Sistema Nacional de Gestão do Patrimônio Genético e do Conhecimento Tradicional Associado (SISGEN - A96FF09). The distribution data was obtained from the analysis of scientific collection materials and previous literature (Bertaco et al., 2016; Dagosta e Depinna 2021).

**TABLE 1:** Species, diploid numbers (2n), sex chromosome systems, numbers of individuals cytogenetically analyzed, and the number of individuals sequenced.

Species	2n	Sex chromosomes	Sampling location	Latitude/Longitude	Cytological analysis	Sequence analysis	References
*P. australis*	♀♂40	Homomorphic	Barra do Bugres—MT	-15.04275,-57.11054	18♂ 30♀	6	Moraes et al. (2017)
*P. brevis*	♀♂42	Homomorphic	Adolpho Ducke Reserve—AM	-2.58207,-59.55530	17♂ 13♀	6	Moraes et al. (2019)
*P. semifasciata*	♀42 ♂41	X_1_X_1_X_2_X_2_/X_1_X_2_Y	Careiro—AM	-3.51000,-60.04000	12♂ 07♀	6	Moraes et al. (2019)
*P. obermulleri*	♀♂42	Homomorphic	Tefé—AM	-3.25507,-64.44548	21♂ 12♀	6	Moraes et al. (2021)
*P. marilynae*	♀♂32	Homomorphic	Ipiranga do Norte—MT	-11.36020,-55.56270	14♂ 08♀	6	Moraes et al. (2021)

Abbreviations: AM, Amazonas; MT, Mato Grosso Brazilian states. Chromosomal data refer to previous studies, according to references.

### DArTseq Sequencing Procedure and Data Filtering

Liver tissue from all individuals was used to perform DArTseq sequencing procedures at Diversity Arrays Technology Pty Ltd. This sequencing method uses a combination of restriction enzymes (SbfI and PstI) that enrich hypomethylated regions (Kilian et al., 2012). Sequencing of the obtained libraries was carried out on the Illumina HiSeq 2500 platform.

The raw data were processed in two different ways to generate the following datasets (I) a matrix of SNPs using Ipyrad v. 0.9.84 (Eaton and Overcast, 2020) and (II) a phased sequence file obtained with pyRAD v3.0.66 (Eaton, 2014). In both, the sequencing adapters were trimmed, and all sequences shorter than 35 base pairs or presenting more than five low-quality bases (Q < 20) were not considered. In dataset I, a single SNP per locus was selected, to reduce the inclusion of linked SNPs, and the SNPs were coded as 0 for reference state homozygotes, 1 for heterozygotes, and 2 for alternative state homozygotes.

The raw data is available in the database of the National Center for Biotechnology Information, whose project access code is PRJNA804560 (access link: https://www.ncbi.nlm.nih.gov/Traces/study/?acc=PRJNA804560&o=acc_s %3Aa). Additionally, datasets one and two are available at https://github.com/PHnarciso/Dataset-1-and-2---Manuscript-*Pyrrhulina*.

### Assessment of Markers Under Selection

A BayeScan analysis was carried to search for potential markers under selection in both datasets (Foll and Gaggiotti, 2008). We performed a total of 5,000 MCMC (Markov Chain Monte Carlo) runs and thinning of 10, with a prior odds value of 100. Values of False Discovery Rate (FDR) were used to classify outliers, and only loci with values lower than 0.01 were considered outliers and removed from subsequent analysis.

### Genetic Diversity

Using dataset II, an analysis was performed in the DnaSP software v. 6.12.03 (Rozas et al., 2019), to obtain the haplotype diversity (*Hd*), Tajimaʼs D (*D*), and two measures of nucleotide diversity per site, *π* and Watterson’s theta (*θw*). Differentiation was also estimated from the nucleotide divergence between samples using the average number of nucleotide substitutions per site between pairs of samples from different species (*Dxy*) and the net divergence, corrected for variation within the samples analyzed (*Da*).

### Genetic Structure and Analysis of Molecular Variance (AMOVA)

A principal coordinate analysis (PCoA) in the R package dartR (Gruber et al., 2018) was used to investigate the genetic structure among species. The genetic structure was also investigated with fastSTRUCTURE (Raj et al., 2014), using the “Lizards-are-awesome” pipeline (Melville et al., 2017). We used the chooseK.py command to select the number of clusters that maximizes likelihood and is more informative for the structure from our dataset. The results from fastStructure were visualized with Clumpak (Kopelman et al., 2015). Additionally, an analysis of molecular variance (AMOVA) was also performed using dataset I, with samples grouped as follows: 1) by species, 2) by the clusters from the best K value on fastStructure, 3) by the clustering pattern generated on the PCoA, and 4) by the presence or absence of the multiple sex chromosome system.

### Species Tree

We used the package SNAPP in BEAST 2.6.4 (Bouckaert et al., 2014) to infer the species tree topology. We included only polymorphic sites, used the default prior for coalescence rate, and calculated backwards and forward substitution rates (parameters u and v) based on the empirical data. We applied a chain length of two million generations, sampling every 5000 iterations. Convergence was evaluated in Tracer 1.7.1 (Rambaut et al., 2018). We obtained the MCC tree based with common ancestor heights in TreeAnnotator, with the first 25% generated trees discarded as burn-in. We exported the consensus tree in FigTree 1.4.4.

### Analysis of Introgression

We assessed admixture between lineages by calculating Patterson’s D-statistics (ABBA-BABA test) in Ipyrad v. 0.9.84 (Eaton and Overcast, 2020) using pooled SNP frequencies of individuals from each species (Durand et al., 2011). We performed 4-taxon tests according to the recovered SNAPP topology (Figure 1), using either *P. marilynae* or *P. australis* as the outgroup, *P. brevis* as P_3_ and *P. semifasciata* and *P. obermulleri* as either P_1_ or P_2_. Significance was measured with 1,000 bootstrap replicates by resampling loci with replacement (following Eaton and Ree, 2013). Results were considered significant according to their Z-scores (significant values >3), which quantifies the number of bootstrap standard deviations in which the D-statistic values deviates from its expected value of zero.

## Results

### Sequencing, Filtering, and Detection of Markers Under Selection

Sequencing of DArTSeq resulted in two million reads per sample, on average, a total of 5,149 SNP-markers were obtained after performing all filtering steps in dataset I, and a total of 5,604 loci were obtained in dataset II. According to the BayeScan result, none of the loci were potentially under selection, as all FDR values were higher than 0.65 and 0.97 for dataset I and dataset II respectively. Thus, all markers were maintained in subsequent analyses.

### Genetic Diversity and Structure

In general, all species showed low diversity values. Values of *π* and *θw* were closely related across all sampled groups, with slightly higher estimates in *P. obermulleri* and *P. semifasciata*, while *P. marilynae* showed the lowest values. (Table 2).

**TABLE 2:** Estimated genome-wide genetic diversity of genus *Pyrrhulina* by species.

Species	Sample size	*Hd*	*π*	*θw*	*D*
*P. australis*	6	0.1230	0.0024	0.0026	−0.2004
*P. brevis*	6	0.1048	0.0020	0.0024	−0.3512
*P. obermulleri*	6	0.2033	0.0042	0.0044	−0.1656
*P. marilynae*	6	0.0338	0.0007	0.0006	0.1380
*P. semifasciata*	6	0.1712	0.0033	0.0039	−0.4412

Abbreviations: *Hd* - haplotype diversity; *π* ***-*** nucleotide diversity; *θw* ***-*** Watterson theta per site and *D* - Tajimaʼs D.

**FIGURE 2:**
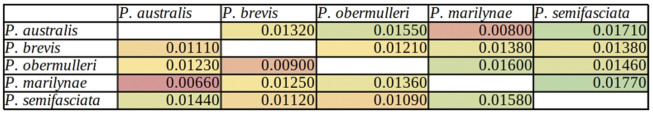
Pairwise *Dxy* per Species is represented in the upper diagonal, and *Da* in the lower diagonal. Higher values are shown in green, and as the values decrease the color changes to yellow, orange, and then red for lower values.

The nucleotide divergence (*Dxy* values) and net divergence (*Da* values) estimates between individuals sampled of different species ranged from 0.01770 to 0.00660 as shown in Figure 2. The lowest divergence values estimates were presented for *P. australis* and *P. marilynae*, and the highest values were observed for comparisons of *P. semifasciata* with these species.

**FIGURE 3:**
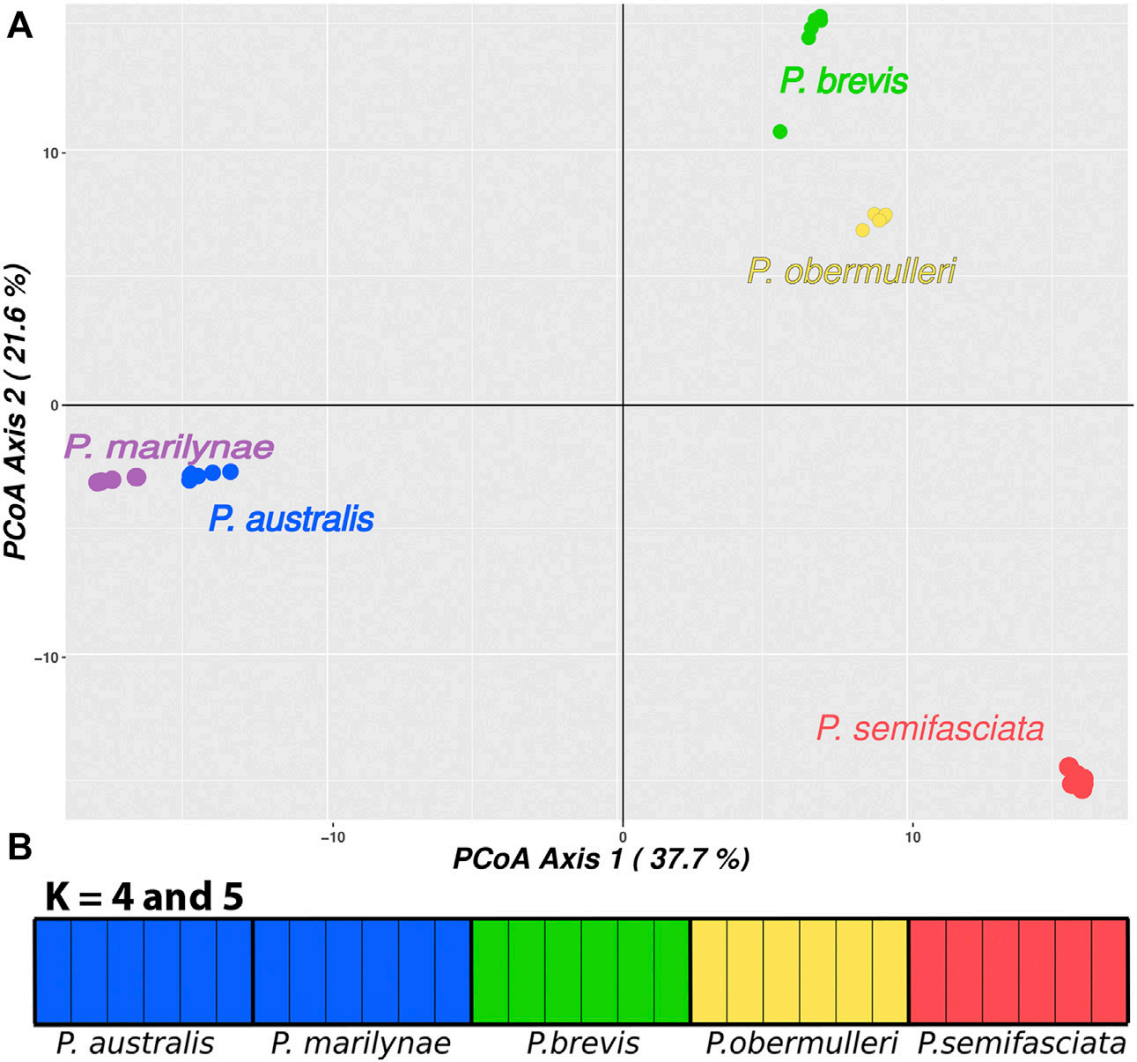
**(A)** Genetic diversity in *Pyrrhulina* species according to a PCoA. The PCoA recovered 37.7% of the total variation in the first principal coordinate (PC1), and 21.6% in the second principal coordinate (PC2). **(B)**—Results for fastStructure with *K* = 4 and *K* = 5. Each vertical bar represents an individual; the bar colors represent the group in which the fastStructure classified the individual; the legend below indicates their species.

The PCoA results separate all species and indicate a higher similarity for species in the same regions, as *P. brevis* and *P. obermulleri* are more similar to each other, as are *P. australis* and *P. marilynae.* In turn, *P. semifasciata* appeared as the most different species of the group (Figure 3).

Results from fastStructure suggested K = 4 as the number of clusters that maximizes both likelihood and is more informative for genetic structure, being the one that best explains the complexity of the data. It is worth mentioning that the clusterization presented in K = 5 is identical to that presented by K = 4, with both results combining *P. australis* and *P. marilynae*. AMOVA results suggested that the clustering strategy presenting the highest variation between groups and the lowest variation within groups was achieved when each separate species was considered (83.66% variation between groups), followed by the fastStructure K = 4 result and by the PCoA (Table 3).

**TABLE 3:** AMOVA percentage of variation within and between each of the tested groups: 1) by species, 2) by the best K in the fast structure, 3) by the groups generated in PCoA, and 4) by the presence or absence of sex chromosomes.

Clustering	Species	FastStructure K = 4	PCoA	Presence/absence of sex chromosomes
Variation between groups	83.664	73.916	61.593	46.534
Variation within group	2.016	12.465	25.087	41.805

### Species Tree and Analysis of Introgression

The species tree recovered in SNAPP showed a relationship between species similar to the structure shown in PCoA (Figure 3), showing *P. australis* and *P. marilynae* as sister species. A sister species relationship was also recovered for *P. semifasciata* and *P. obermulleri*, with *P. brevis* closer to this last two (Figure 1). Results of the admixture tests using D-statistics were not able to detect significant introgression in *P. semifasciata* or *P. obermulleri*, regardless of whether *P. marilynae* or *P. australis* were considered as the outgroup (Table 4). This result agrees with the lack of admixture suggested by fastSTRUCTURE (Figure 3).

**TABLE 4:** Results of the ABBA-BABA test for admixture assessment.

Outgroup	P_3_	P_2_	P_1_	D	Z	ABBA	BABA
*P. marilynae*	*P. brevis*	*P. obermulleri*	*P. semifasciata*	−0.0214164	0.44500958	180.753565	188.665185
*P. australis*	*P. brevis*	*P. obermulleri*	*P. semifasciata*	0.00523111	0.1052923	182.665167	180.764028
*P. marilynae*	*P. brevis*	*P. semifasciata*	*P. obermulleri*	−0.0052311	0.1035013	180.764028	182.665167
*P. australis*	*P. brevis*	*P. semifasciata*	*P. obermulleri*	−0.0052311	0.10302304	180.764028	182.665167

Abbreviations: D, overrepresentation of ABBA against BABA patterns as measured by the D-statistics; Z, Z-score test to assess whether the D-statistics is significantly (Z > 3) different from zero; ABBA, frequency of ABBA patterns; BABA, Frequency of BABA patterns.

## Discussion

Here the first genomic evaluation of five *Pyrrhulina* species was performed. Our results highlight that genetic diversity among the analyzed *Pyrrhulina* species is consistent with their chromosomal features. In addition, evidence was found that the occurrence of chromosomal rearrangements including variation in sex chromosome systems might have contributed to increase differentiation.

Overall, the genetic diversity indexes (*π; θ*_*W*_) (Table 2) were low, being the highest ones observed in *P. obermulleri* and *P. semifasciata* and the lowest in *P. marilynae* followed by *P. brevis*. Low diversity values are somewhat expected due to the nature of our sequencing technique (see methods) and similar results were found in populations from *Hoplias malabaricus* under the same sequencing technique (Souza et al. submitted). According to *Dxy* and *Da* values (Figure 2), *P. semifasciata* and *P. marilynae* are very distinct from each other, presenting the highest observed values (0.01770 and 0.0158). They are also distinct morphologically (Netto-Ferreira and Marinho, 2013) and present non-overlapping distributions in the Amazon basin. *Pyrrhulina semifasciata* occur in the Purus, Juruá, Negro, Branco rivers, Amazonas main channel (Dagosta and De Pinna, 2021), and Madeira River while *P. marilynae* is restricted to the upper portions of Tapajós and Xingu (Netto-Ferreira & Marinho, 2013) (Figure 1). Similarly, values of *Dxy* and *Da* between *P. semifasciata* and *P. australis* are also very high (0.0171 and 0.0144), indicating their genetic distinctiveness. Furthermore, their distribution does not overlap (Figure 1) and they are also quite distinct morphologically and probably distantly related species, according to a morphology-based phylogenetic analysis (Marinho, 2014). This results are also in agreement with the species tree, as *P. marilynae* and *P. australis* were recovered as sister species in a distinct clade (Figure 1). However, it is important to emphasize that a more comprehensive species-level sampling is still necessary before claiming sister species relationships, especially in taxa that are apparently divergent such as *P. brevis* and *P. obermulleri.*

*Pyrrhulina semifasciata*, the only species in the family up to now analyzed with heteromorphic sex chromosomes, presents high genetic differentiation values when compared to all other species. This could suggest that the presence of such multiple sex system promoted genetic differentiation in the species. The association of sex chromosomes and a high genetic differentiation was also evidenced in previous studies for some populations of other species such as the wolf fishes *Hoplias malabaricus* (Souza et al., submitted) and *Erythrinus erythrinus* (Souza et al., 2022). In addition, other studies suggest that an event of sex chromosome turnover in plants could have promoted the reduction of gene flow between groups of *Rumex hastatulus*, which could have contributed to further accumulation of genetic differences (Beaudry et al., 2019). Furthermore, the emergence of a neo-sex chromosome system is suggested as a factor that may have driven the speciation process in sticklebacks (Kitano et al., 2009). Therefore, the emergence of sex neo-chromosomes could result in a rapid evolutionary mechanism fixing genetic variation in different species (Beaundry et al., 2019). Indeed, our results suggest that *P. semifasciata*, which was collected in syntopy with *P. brevis* and presents a different chromosomal structure with a multiple sex chromosome system, is also genetically highly divergent from that latter species.

Therefore, we present three main hypotheses for the observed scenario (I) *P. brevis* and *P. semifasciata* evolved in allopatry. Later, their distribution overlapped and the current genetic differences are the result of this previous allopatric period and not related to the sex chromosomes emergence (II) these species evolved in allopatry and the sex chromosomes present in *P. semifasciata* may have acted as an additional barrier to introgression, fostering their genetic differentiation after they became sympatric, and (III) the emergence of a multiple sex chromosome system of the X_1_X_2_Y-type in *P. semifasciata* have acted as key factor to reduction of the gene flow and contributed to the speciation process and the significant genetic differentiation observed for this species.

The PCoA result also suggested that *P. semifasciata* stayed isolated from all other species, while *P. brevis* clustered with *P. obermulleri* (Figure 3). Many works highlight the important role of neo-sex chromosomes in preventing introgression, which is agreement with the lack of introgression between sympatric species detected here (Table 4), due to pairing problems associated with meiotic segregation of heterozygous rearrangements (e.g., McKee et al., 1998; Kitano and Peichel, 2012; Beaudry et al., 2019). Similarly, sex chromosomes might promote speciation, as some genes that play a role in reproductive isolation between populations can be accumulated in neo-sex chromosomes (Reinhold, 1998; Lindholm and Breden, 2002; Kitano and Peichel, 2012). Haldane’s rule postulates that hybrids of the heterogametic sex from closely related species are often sterile, thus hybrid incompatibility also may act as a reproductive isolating barrier and contribute to prevent introgression (Haldane, 1922; Coyne and Orr, 2004). In both scenarios, the neo-sex chromosomes system could have contributed to genetic differentiation or speciation. Hence, it may be possible that the chromosomal rearrangements present in *P. semifasciata* may have had an impact on the diversity of *Pyrrhulina* species.

In contrast, some species showed low differentiation, indicating that they are more similar genetically. The *Dxy* and *Da* values between *P. marilynae* and *P. australis* were the lowest ones (0.008 and 0.0066 respectively) and these two species were recovered as sister species in our phylogenetic analysis (Figure 1). Also, both species share with *P. vittata* a series of unique morphological characters which indicate that they form a monophyletic clade (Netto-Ferreira and Marinho, 2013). *Pyrrhulina brevis* and *P. obermulleri* presented low *Dxy* and *Da* values as well (0.0121 and 0.0090) in addition to be close to each other in the PCoA analysis (Figure 3) and be recovered in the same clade in the species tree, although not as sister species (Figure 1). All Tajima’s D values were negative except for *P. marilynae.* Negative results may suggest a recent event of population expansion, or that purifying selection is acting mainly in *P. semifasciata* and *P. brevis*. The small positive value presented by *P. marilynae* could indicate a bottleneck event, but this seems unlikely due to the low value.

The results of PCoA indicated a clear clustering of species sampled in the same geographical region, except for *P. semifasciata*, which was collected in sympatry with *P. brevis* and did not cluster with any other species (Figure 3). In fastStructure K = 4 result, only *P. australis* and *P. marilynae* were grouped. In both fastStructure and PCoA results, *P. australis* and *P. marilynae* were closely related, and such a connection is also presented in the speciestree (Figure 1) and supported by the species tree presented by Marinho (2014).

In addition, AMOVA results suggest that grouping individuals by species maximize the variation between groups, although the groups generated by fastStructure and PCoA have also shown informative values (Table 3). Therefore, AMOVA results show that the clustering of individuals by species best explains the complexity of the data and therefore suggests that the species are well separated. The relationships between river reorganizations and biodiversity are a complex issue, as previously isolated habitats and populations can be merged, increasing dispersal and local diversity, and reducing speciation and extinction rates. They can also separate previously connected environments, leading to increased genetic isolation and speciation, and extinction rates (Tagliacollo et al., 2015; Albert et al., 2018). Therefore, the complex biogeographic history of the region complicates the understanding of *Pyrrhulina* genetic diversity considering that the current geographic distribution of species may not be the same as in the past and a comprehensive species-level phylogeny is still necessary to infer past geographic distributions.

It is widely known that chromosomal rearrangements might have a role in speciation (Rieseberg, 2001; Navarro and Barton, 2003; Basset et al., 2008). Chromosomal rearrangements have the potential to limit introgression, thus facilitating the origin and maintenance of reproductive isolation through recombination suppression (Faria and Navarro, 2010; Sichová et al., 2015). Here, *P. australis* and *P. marilynae* were recovered as sister species in our phylogenetic analysis. This suggests a low genetic difference despite their different chromosomal formulae. Conversely, the species *P.* semifasciata, the only with a multiple sex chromosome system, showed high differentiation to all other species. This furnishes additional evidence for the role of chromosomal rearrangements, especially those associated with sex chromosomes, as potential important reproductive barriers between these species.

## Conclusion

Our results highlighted the distribution of the genetic diversity among the analyzed *Pyrrhulina* species and contributed to our understanding of their evolutionary history. However, the scarcity of phylogenetic data involving all *Pyrrhulina* species does not permit us to do a cause-and-effect correlation. Besides, the occurrence of chromosomal rearrangements leading to the presence of the X_1_X_2_Y sex chromosome system in *P. semifasciata* could have led to an intensification of the genetic differentiation of this species by preventing introgression with the other species. Future work with a better population and species-level sampling is still needed to bring new insights to 1) species delimitation, 2) infer demographic trends, 2) infer past distributional ranges, and 4) study the tempo and mode of speciation, including chromosomal rearrangements as a mechanism for isolation.

## Data Availability

The datasets presented in this study can be found in online repositories. The names of the repository/repositories and accession number(s) can be found below: SRA, PRJNA804560.
